# Unique genome organization of non-mammalian papillomaviruses provides insights into the evolution of viral early proteins

**DOI:** 10.1093/ve/vex027

**Published:** 2017-10-06

**Authors:** Koenraad Van Doorslaer, Valeria Ruoppolo, Annie Schmidt, Amelie Lescroël, Dennis Jongsomjit, Megan Elrod, Simona Kraberger, Daisy Stainton, Katie M Dugger, Grant Ballard, David G Ainley, Arvind Varsani

**Affiliations:** 1 ACBS and Bio5, University of Arizona, 1657 E Helen St, Tucson, AZ 85721, USA; 2 Laboratório de Patologia Comparada de Animais Selvagens (LAPCOM), Departamento de Patologia, Faculdade de Medicina Veterinária e Zootecnia, Universidade de São Paulo, São Paulo, Brazil; 3 Point Blue Conservation Science, Petaluma, CA 94954, USA; 4 Centre d'Ecologie Fonctionnelle et Evolutive – CNRS, UMR 5175, Montpellier, France; 5 The Biodesign Center for Fundamental and Applied Microbiomics, Center for Evolution and Medicine, School of Life sciences, Arizona State University, Tempe, AZ 85287, USA; 6 School of Biological Sciences, The University of Queensland, Brisbane, QLD, Australia; 7 U.S. Geological Survey, Oregon Cooperative Fish and Wildlife Research Unit, Department of Fisheries and Wildlife, Oregon State University, Corvallis, OR 97331, USA; 8 HT Harvey and Associates, Los Gatos, CA 95032, USA; 9 Structural Biology Research Unit, Department of Clinical Laboratory Sciences, University of Cape Town, Observatory, Cape Town, 7925, South Africa

**Keywords:** papillomavirus, evolution, bird, reptile, avian

## Abstract

The family *Papillomaviridae* contains more than 320 papillomavirus types, with most having been identified as infecting skin and mucosal epithelium in mammalian hosts. To date, only nine non-mammalian papillomaviruses have been described from birds (*n* = 5), a fish (*n* = 1), a snake (*n* = 1), and turtles (*n* = 2). The identification of papillomaviruses in sauropsids and a sparid fish suggests that early ancestors of papillomaviruses were already infecting the earliest Euteleostomi. The Euteleostomi clade includes more than 90 per cent of the living vertebrate species, and progeny virus could have been passed on to all members of this clade, inhabiting virtually every habitat on the planet. As part of this study, we isolated a novel papillomavirus from a 16-year-old female Adélie penguin (*Pygoscelis adeliae*) from Cape Crozier, Ross Island (Antarctica). The new papillomavirus shares ∼64 per cent genome-wide identity to a previously described Adélie penguin papillomavirus. Phylogenetic analyses show that the non-mammalian viruses (expect the python, *Morelia spilota*, associated papillomavirus) cluster near the base of the papillomavirus evolutionary tree. A papillomavirus isolated from an avian host (Northern fulmar; *Fulmarus glacialis*), like the two turtle papillomaviruses, lacks a putative E9 protein that is found in all other avian papillomaviruses. Furthermore, the Northern fulmar papillomavirus has an E7 more similar to the mammalian viruses than the other avian papillomaviruses. Typical E6 proteins of mammalian papillomaviruses have two Zinc finger motifs, whereas the sauropsid papillomaviruses only have one such motif. Furthermore, this motif is absent in the fish papillomavirus. Thus, it is highly likely that the most recent common ancestor of the mammalian and sauropsid papillomaviruses had a single motif E6. It appears that a motif duplication resulted in mammalian papillomaviruses having a double Zinc finger motif in E6. We estimated the divergence time between Northern fulmar-associated papillomavirus and the other Sauropsid papillomaviruses be to around 250 million years ago, during the Paleozoic-Mesozoic transition and our analysis dates the root of the papillomavirus tree between 400 and 600 million years ago. Our analysis shows evidence for niche adaptation and that these non-mammalian viruses have highly divergent E6 and E7 proteins, providing insights into the evolution of the early viral (onco-)proteins.

## 1. Introduction

The family of the *Papillomaviridae* contains more than 320 distinct viral species. In recent years, there has been an exponential increase in the identification of non-human-associated papillomaviruses. The majority of known papillomaviruses have been isolated from humans and other mammalian hosts. To date, nine non-mammalian viruses have been described. These viruses infect birds including, yellow-necked francolin (*Francolinus leucoscepus*), common chaffinch (*Fringilla coelebs*), northern fulmar (*Fulmar glacialis*), African Grey parrot (*Psittacus erithacus*) and Adélie penguin (*Pygoscelis adeliae*) (Terai et al. 2002; Tachezy et al. 2002; Van Doorslaer et al. 2009; Varsani et al. 2014; Gaynor et al. 2015; Varsani et al. 2015 ), as well as green sea turtle (*Chelonia mydas*), loggerhead sea turtle (*Caretta caretta*) (Herbst et al. 2009), Carpet python (*Morelia spilota*) (Lange et al. 2011), and gilt-head bream fish (*Sparus aurata*) (Lopez-Bueno et al. 2016).

Papillomaviruses had been thought to be restricted to amniotes (Rector and Van Ranst 2013). However, recent studies that have identified papillomaviruses in fish (Lopez-Bueno et al. 2016) reveal that early ancestors of the papillomaviruses were already infecting the earliest members of Euteleostomi. That clade includes more than 90 per cent of the living vertebrate host species, and progeny viruses could have been passed on to all members of this clade, inhabiting virtually every habitat on the planet. Also, the hosts that have been extensively studied (humans, cattle, dogs, etc.), have an extensive repertoire of highly species-specific papillomaviruses. The observation that papillomaviruses cause benign infections unable to cross the hosts' species-barrier has led to the hypothesis that papillomaviruses coevolved alongside their hosts (Bernard 1994). However, the papillomavirus phylogeny shows evidence of initial, ancestral niche sorting events followed by virus–host linked speciation (Shah et al. 2010; Gottschling et al. 2011; Van Doorslaer 2013). This suggests that the acquisition of new ecological niches on the host (gain/loss of fur, the evolution of sweat glands, etc.), not host speciation per se may be driving papillomaviruses evolution (Brooks and Ferrao 2005).

As part of this study, we report the identification of a novel papillomavirus from an Adélie penguin from Cape Crozier, Ross Island (Antarctica). We show that this novel virus is most closely related to a previously identified Adélie penguin papillomavirus and that together they cluster with the other non-mammalian viruses near the base of the papillomavirus evolutionary tree. Comprehensive analysis of the ten non-mammalian viral genomes provides evidence for niche adaptation followed by co-evolution of these papillomaviruses. Furthermore, these non-mammalian viruses have highly divergent E6 and E7 proteins, consideration of which allowed for a deeper understanding of the evolutionary history of these papillomaviruses. The E6 and E7 protein’s unique features may enable a better understanding of the evolution of these oncoproteins.

## 2. Materials and methods

### 2.1 Ethics statement

The samples were collected under Oregon State University’s Institutional Animal Care and Use Committee approved Animal Care and Use Protocol (ACUP permit #s 4130 and 4622) Antarctic Conservation Act Permit #2011-002 M#7 from the National Foundation of Science (USA) through H.T. Harvey & Associates.

### 2.2 Sample preparation, DNA enrichment, and sequencing

The study was conducted at the Cape Crozier Adélie penguin colony (77°27′S, 169°12′E), Ross Sea, Antarctica. All cloacal samples were taken under the auspices of the long-term research program in place at Cape Crozier since the 1996–97 austral summer which individually marked a sample (500–1,000) of near-fledglings with numbered stainless-steel flipper bands each year.

Cloacal swabs of twenty-five birds ranging 7–18 years of age (eight females and seventeen males) were taken in December 2014 (during the breeding season) and stored in UTM™ Viral Transport Media (Copan). UTM™ Viral Transport Media (1000 µl) was filtered through 0.2 µm syringe filters, and 200 µl of this was subsequently used to extract viral DNA using the High Pure Viral Nucleic Acid Kit (Roche Diagnostics). Viral DNA (1 µl) from each sample was used to amplify circular DNA molecules using rolling-circle amplification (RCA) with the TempliPhi™ kit (GE Healthcare). The RCA DNA (5 µl aliquot from each sample) was pooled and sequenced on an Illumina 4000 (Illumina) sequencer at Macrogen Inc. (Korea).

The paired-end reads were *de novo* assembled using ABySS v1.9 (Simpson et al. 2009) and contigs >750 nucleotides were analyzed using BLASTx against a local viral database. A contig of 7,717 nucleotides was identified that encodes proteins that are homologous to those of papillomavirus. Further analysis of this contig revealed a 63  nucleotide terminal repeat and with the repeated region removed this contig represented a unit length papillomavirus. A pair of abutting primers (AdP2F 5'- CCA TGA GAA ACC TTT TAG TCT TCC CGG G-3' and AdP2R 5'-GGT ATG AAA GAG CCA GTG TCA GGC TTA TTG-3') were designed in the minor capsid protein (L2) open reading frame (ORF) to screen and recover the full genome of this papillomavirus. The RCA product (0.5 µl) from the viral DNA from the cloacal swab samples was used as a template for polymerase chain reaction (PCR) to screen individual Adélie penguin samples for the papillomavirus with the AdP2F/R primers with the following thermal cycling protocol with Kapa HiFi Hotstart DNA polymerase: 95 °C for 3 minutes; twenty-five cycles of 98 °C for 20 seconds, 60 °C for 15 seconds, 72 °C for 8 minutes, and a final extension of 72 °C for 8 minutes followed by a cooling step to 4 °C for 10 minutes. The PCR products were resolved on a 0.7 per cent agarose gel stained with SYBR safe, and the ∼7.5 kb amplicon was excised, purified, and cloned into the pJET1.2 plasmid vector (ThermoFisher), and Sanger sequenced by primer walking at Macrogen Inc. (Korea). The Sanger sequencing contigs were assembled using DNA Baser (Heracle BioSoft S.R.L. Romania).

### 2.3 Sequence analysis

Viral ORFs were predicted using a custom, papillomavirus-specific annotation tool as described previously (Van Doorslaer et al. 2013). Genome-wide identity analysis was performed using default settings within SDT v1.2 (Muhire et al. 2014). Pairwise sequence comparisons with PaPV1 (Varsani et al. 2014) were performed within Geneious version 10.2 (Kearse et al. 2012). The annotated sequence of PaPV1 was downloaded from the PaVE database (Van Doorslaer et al. 2013; Van Doorslaer et al. 2017). The L-INS-I algorithm within MAFFT v7.3 (Katoh et al. 2002; Katoh and Standley 2013) was used to align the translated protein sequence for each individual ORF (using the Blosum62 scoring matrix). Translated protein sequences were back translated to obtain codon-conserving nucleotide alignments. To aid in L1-based classification, the L1 ORF located between nucleotides 6,114 and 7,637 (PaPV1) and 6,116 and 7,654 (PaPV2) was used. The sequence of novel papillomavirus from Adélie penguin is deposited on GenBank under accession number MF168943.

### 2.4 Maximum likelihood phylogenetic tree construction

For genera containing more than five virus types, five viruses were randomly chosen to represent the genus, for other genera all virus sequences were included. Unclassified viruses, i.e. viruses not yet officially assigned to a genus by the ICTV, were excluded from the analysis. The sequences for the viral E1, E2 and L1 proteins of the 120 viruses were downloaded from PaVE (Van Doorslaer et al. 2013; Van Doorslaer et al. 2017). Individual proteins were aligned using MAFFT v7.3 (Katoh et al. 2002; Katoh and Standley 2013), E1 and E2 were aligned using the E-INS-I algorithm, G-INS-I was used for the L1 alignment. Aligned sequences were concatenated. A partitioning scheme allowing each protein to evolve according to the LG + I+G + F model of evolution was identified to be the best fit under the corrected Akaike information criterion (AICc) as implemented within PartitionFinder (Guindon et al. 2010; Lanfear et al. 2017). Maximum likelihood (ML) trees were estimated using RAxML-HPC v.8 on XSEDE (Miller et al. 2015), performing a rapid bootstrap analysis followed by a search for the best tree. The number of bootstrap replicates (*n* = 108; Weighted Robinson-Foulds = 2.46) were determined according to the autoMRE criteria implemented in RAxML (Pattengale et al. 2010; Stamatakis 2014). The resulting phylogenetic tree displayed represents the best ML tree.

### 2.5 Evolutionary hypothesis testing

Four distinct evolutionary hypotheses were compared (phylogenetic trees in Newick format are available on FigShare). The constrained trees were estimated using the same settings as used for the non-constrained ML tree (previous paragraph). The RAxML calculations started with an incomplete constraint, i.e. only the relevant nodes were constrained while the position of the remaining taxa was optimized under ML respecting the given constraints. Site-likelihoods associated with each tree were calculated using Tree-Puzzle (Schmidt et al. 2002) and compared using different hypothesis testing methods implemented in CONSEL (Shimodaira and Hasegawa 2001). The tests implemented are based on the RELL bootstrapping method. Hence, the parameters and branch lengths estimated by RAxML are used for the analysis. A significance limit of α* *=* *0.05 was used for all analyses.

### 2.6 Dating of the FgPV1 divergence

The E1, E2, and L1 protein sequences were aligned as described above. The divergence times were estimated using an uncorrelated relaxed clock (Drummond et al. 2006) with a calibrated Yule speciation model (Heled and Drummond 2012) in BEAST 2.4 (Le and Gascuel 2008; Heled and Drummond 2012; Bouckaert et al. 2014) as implemented on CIPRES (Miller et al. 2015). The partitioning scheme and evolutionary model were the same as in that used in evolutionary hypothesis testing described above. Site models and phylogenetic trees were estimated for the entire alignment. An uncorrelated relaxed clock model was assigned to each Individual protein. The previously estimated ML tree was used as a starting tree, with monophyletic requirements imposed on the calibration nodes. Lognormal priors were used to calibrate nodes 2, 9, 25, and 26 based on the divergence estimates of the hosts. Calibration means and 95 per cent confidence interval were downloaded from TimeTree (www.timetree.org; Hedges et al. 2006; Hedges et al. 2015). The MCMC chains were run for 1 × 10^8^ generations. Samples were collected every 5,000th generation. The analysis was repeated for a total of six independent runs. The resulting log files and trees were combined using LogCombiner and resampled (every 10,000th generation) while discarding a 20 per cent burn-in. An estimated sample size of >200 was achieved for most parameters. The reported estimates (Fig. 2; Supplementary Table S1) are based on the combined dataset.

A posterior distribution of potential phylogenetic trees for the E1–E2–L1 concatenated alignment described above was estimated using MrBayes 3.2.6 (Huelsenbeck and Ronquist 2001; Ronquist and Huelsenbeck 2003; Ronquist et al. 2012; Miller et al. 2015). Two chains of MCMC were run for 1,000,000 steps, and parameters were logged every 1,000 steps with the initial 25 per cent discarded as burn in. Chain convergence was confirmed by comparing different parameter’s potential scale reduction factors (∼1.000), by analyzing individual traces in Tracer v1.6 (http://beast.bio.ed.ac.uk/Tracer) and by using AWTY (Nylander et al. 2008). The analysis was repeated three separate times, and post burn-in samples were combined. The time-dependent rate phenomenon can be modeled using a power-law rate-decay model (T = αS^β^). For each of the estimated posterior Bayesian phylogenies (*n* = 5905), a set of patristic distances (S) was calculated starting from either PePV1 or CPV6. Matching divergence time estimates (T) associated with the host species were downloaded from TimeTree (www.timetree .org) (Hedges et al. 2006; Hedges et al. 2015). The values of S and T were log-transformed (base 10), and the average ±SD was plotted. Generalized Deming regression (Ripley and Thompson 1987) was performed using the Deming function in R 3.2.1 (Team RC 2014). The linear model was then extrapolated to estimate the timescale of other nodes from their S estimates. Data, Python, and R scripts to perform these analyses are available through FigShare. 

## 3. Results

### 3.1 Identification of a novel papillomavirus isolated from an Adélie penguin

One of the twenty-five cloacal swab samples of Adélie penguins from Cape Crozier on Ross Island (16-year-old female) was positive for a novel papillomavirus. This novel papillomavirus shares ∼64 per cent genome-wide sequence identity with Pygoscelis adeliae papillomavirus 1 (PaPV1), which was identified from a fecal sample retrieved at Cape Crozier (Varsani et al. 2014). Based on the recommendation by the animal papillomavirus reference center (Bernard et al. 2010), the novel papillomavirus is designated Pygoscelis adeliae papillomavirus type 2 (PaPV2).

**Figure 1.**
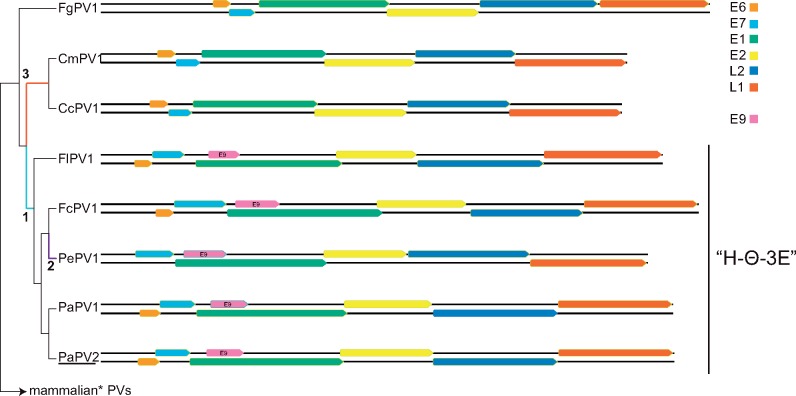
Genomic organization of Sauropsid papillomaviruses. Illustration of the genomic organization of the known bird and turtle papillomaviruses, i.e. Caretta caretta papillomavirus 1 (CcPV1), Chelonia mydas papillomavirus 1 (CmPV1), Francolinus leucoscepus papillomavirus 1, (FlPV1), Fringilla coelebs papillomavirus 1 (PcPV1), Psittacus erithacus papillomavirus 1 (PePV1), Fulmar glacialis papillomavirus type 1 (FgPV1), and Pygoscelis adeliae papillomavirus 1 and 2 (PaPV1 and PaPV2). PaPV2, the virus isolated as part of this report is underlined. The open reading frames are color coded. Phylogenetic tree on the left shows the evolutionary relationships between the different viruses. Colored/numbered branches refer to “molecular morphology traits”, see main text for details. The mammalian virus clade contains MsPV1, the virus isolated from a lesion on a Carpet python. The ‘H–Θ–3E’ viruses are indicated.

**Figure 2.**
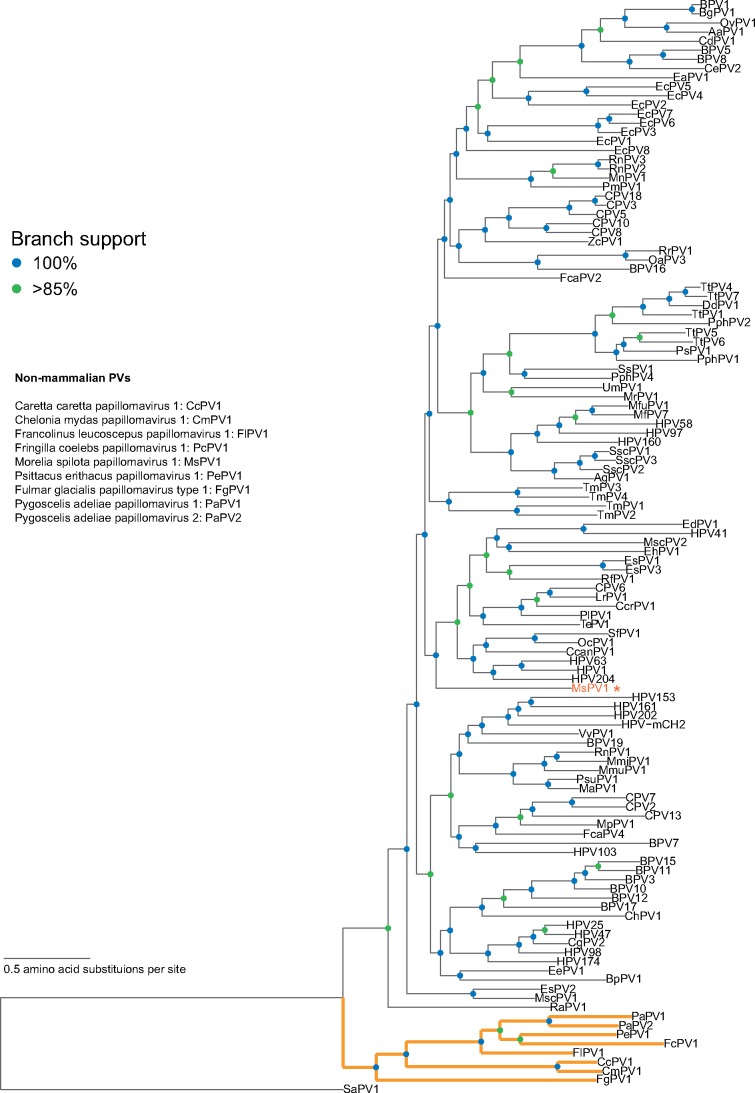
Maximum likelihood phylogenetic tree showing the relationship between the sauropsid and mammalian papillomaviruses. Colored nodes indicate bootstrap support (blue=100%, green >85%). The branches leading to the sauropsid viruses are shown in bold and orange. Despite being isolated from a python, the Morelia spilota papillomavirus type 1 (MsPV1; indicated with an asterisk) clusters with the mammalian viruses. TempEst (Rambaut et al. 2016) was used to identify the root of the tree.

The 7,654-bp double-stranded genome of PaPV2 contains seven ORFs ( E6, E7, E1, E9, E2, L2, and L1) on the same coding strand (Fig. 1). Both pairwise identities and phylogenetic analysis show that PaPV2 is most closely related to PaPV1 (Figs 1 and 2). The L1 gene of PaVP1 and PaPV2 share 62.5 per cent nucleotide sequence identity thus suggesting that both these Adélie penguin-associated papillomaviruses likely belong to the genus *Treisepsilonpapillomavirus* (Table 1).

**Table 1.** Pairwise sequence comparison between PaPV1 and PaPV2.

PaPV1 vs. PaPV2	Nucleotide identity^a^	Amino acid similarity^b^
E6	43.4	60.0
E7	45.3	62.1
E1	63.4	80.2
E9	53.7	79.5
E2	49.8	68.7
L1	62.5	86.2
L2	53.2	74.5

^a^Pairwise, codon conserving aligment.

^b^Blosum62 matrix with threshold = 0.

### 3.2 Phylogenetic analysis clusters mammalian and sauropsid papillomaviruses into separate monophyletic clades

A substantial part of papillomavirus evolutionary history can be explained by virus-host co-speciation (Van Doorslaer 2013). The recent identification of fish papillomaviruses (Lopez-Bueno et al. 2016) extends the evolutionary history of this group of viruses to at least 400 million years ago (MYA) (Osteichthyes and Amniota shared a last common ancestor around 400 MYA (dos Reis et al. 2015). To reconstruct the papillomavirus evolutionary history, we estimated a phylogenetic tree including all known non-mammalian papillomaviruses (including the fish papillomavirus Sparus aurata papillomavirus 1 (SaPV1), bird (*n* = 6), snake (MsPV1; *n* = 1), and testudines (*n* = 2) infecting papillomaviruses), as well as representative papillomaviruses associated with different mammalian hosts (*n* = 109). A ML tree was constructed based on a concatenated protein alignment of E1, E2, and L1 (Fig. 2). Assuming the existence of a molecular clock, the software program TempEst (Rambaut et al. 2016) identified the fish papillomavirus (SaPV1) as the root of the papillomavirus phylogenetic tree. Consistent with the hypothesis of co-speciation, the initial split between the mammalian and non-mammalian viruses matches the equivalent split between their hosts; the papillomavirus tree shows two well-separated, monophyletic clades. The first clade clusters the mammalian viruses, with the turtle and bird viruses clustering in a separate, monophyletic clade. As a notable exception, MsPV1 (the papillomavirus isolated from a *Morelia spilota* (Carpet python) lesion), clusters with the mammalian viruses.

### 3.3 No strict co-speciation within the turtle-bird clade

The phylogenetic tree with the highest likelihood (Fig. 2) suggests that the avian papillomaviruses (*n* = 5) form a paraphyletic group. This reconstruction places the Fulmar glacialis papillomavirus 1 (FgPV1), a virus infecting the Northern Fulmar, sister to a clade containing the turtle and all other avian papillomaviruses. To formally test this hypothesis, we compared different tree topologies using the approximately unbiased (AU) test (Shimodaira 2002) as implemented within CONSEL (Shimodaira and Hasegawa 2001). We tested the following alternative hypotheses: 1, FgPV1 speciation predates divergence of bird and turtle viruses (i.e. the ML solution); 2, all avian papillomaviruses cluster together, separate from the turtle papillomaviruses; 3, two monophyletic clades containing Caretta caretta papillomavirus 1 (CcPV1), Chelonia mydas papillomavirus 1 (CmPV1), and avian papillomaviruses (Francolinus leucoscepus papillomavirus 1, FlPV1; Fringilla coelebs papillomavirus 1, PcPV1; Psittacus erithacus papillomavirus 1, PePV1; FgPV1, PaPV1, and PaPV2), respectively; 4, constraint based on the accepted host phylogeny (Prum et al. 2015). Using an α of 0.05, only the strict host phylogeny constraint (option 4) was rejected as an alternative topology (Table 2). This suggests that the exact phylogenetic position of FgPV1 cannot be determined. In addition, as is the case in the mammalian clade, strict co-speciation within the non-mammalian papillomavirus clade should be rejected in favor of more complex evolutionary history (Van Doorslaer 2013).

**Table 2.** Statistical comparison of four distinct phylogenetic hypotheses.

Phylogenetic tree (newick)	Rank	δ log likelihood to best tree	AU	KH	SH	wKH	wSH
Unconstrained	1	0	0.756	0.636	0.919	0.636	0.928
(SaPV1,(((CcPV1,CmPV1),(**FgPV1**,FlPV1,PePV1,FcPV1,PaPV2,PaPV1)), (mammalian*)));	2	6.6	0.476	0.364	0.805	0.364	0.795
(SaPV1,((**FgPV1**,(CcPV1,CmPV1,FlPV1,PePV1,FcPV1,PaPV2,PaPV1)), (mammalian*)));	3	17.6	0.242	0.205	0.476	0.205	0.394
(SaPV1,(((**FgPV1**,CcPV1,CmPV1),(FlPV1,PePV1,FcPV1,PaPV2,PaPV1)), (mammalian*)));	4	18.4	0.242	0.221	0.488	0.221	0.445
(SaPV1,(((CcPV1,CmPV1),(FlPV1,((FcPV1,PePV1),(**FgPV1**,(PaPV2,PaPV1))))), (mammalian*)));	5	119.2	0.002	0	3.00E-04	0	0

AU, *P*-value for the approximately unbiased test; KH, *P*-value for the Kishino–Hasegawa test; SH, *P*-value for the Shimodaira–Hasegawa test; wKH, *P*-value for the weighted Kishino–Hasegawa test; wSH, *P*-value for the weighted Shimodaira–Hasegawa test.

### 3.4 The E9 protein is a synapomorphy of the H−Θ–3E Clade

Phylogenetic analysis based on three viral proteins did not enable us to determine the exact evolutionary history of FgPV1. However, all tested topologies clustered the other bird viruses (FcPV1, FlPV1, PePV1, PaPV1, and PaPV2 belonging to the *Eta-*, *Theta-*, and *Treisepsilonpapillomavirus* genera) and turtle viruses (CcPV1 and CmPV1; genus *Dyozetapapillomavirus*) into two distinct, monophyletic clades. From here on we refer to the avian viruses in the *Eta-*, *Theta-*, and *Treisepsilonpapillomavirus* monophyletic clade as ‘H−Θ−3E’. To date, H − Θ−3E clusters all known avian papillomaviruses except the fulmar FgPV1 virus.

**Figure 3.**
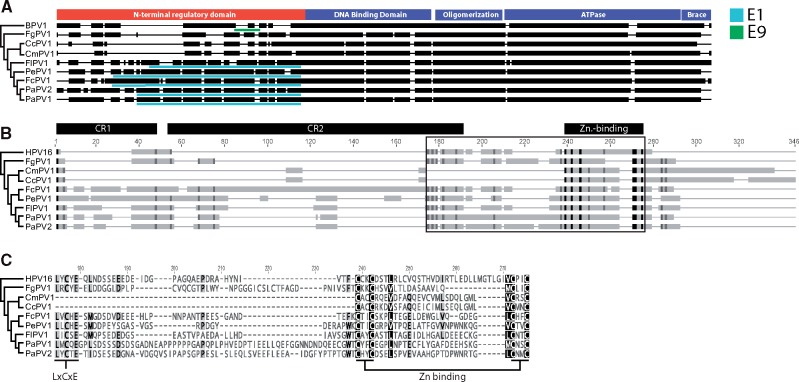
Genetic plasticity in the sauropsid papillomaviruses. (A) The E1 ORF of the sauropsid papillomaviruses and Bovine papillomavirus type 1 (BPV1) were aligned. In this alignment, thin lines indicate indels, while solid black bars indicate confidently aligned regions. The phylogenetic tree indicates the evolutionary history. The bars above the alignment indicate the different E1 protein domains identified in BPV1 (Bergvall et al. 2013). The +1 frame of this alignment was investigated for the presence of a large, conserved ORF known as E9 (light blue bars, Van Doorslaer et al. 2009). The avian viruses in the *Eta-*, *Theta-*, and *Treisepsilonpapillomavirus* genera (‘H–Θ–3E ’) contain this ORF. Like all mammalian viruses, the BPV1 E8 exon is located in a similar position (green bar). (B) The E7 protein of the sauropsid papillomaviruses and human papillomavirus type 16 (HPV16) were aligned. In this alignment, thin lines indicate indels, solid gray bars indicate confidently aligned regions, and black bars indicate identical residues. The phylogenetic tree indicates the evolutionary history. The bars above the alignment indicate the different E7 protein domains identified in HPV16 (Roman and Munger 2013). The rectangle indicates the part of the alignment that is enlarged in C. (C) Amino-acid level view of part of the alignment shown in B. The alignment is shaded by conservation. The conserved LxCxE and Zinc-binding motifs are indicated (Roman and Munger 2013).

Of note, all known members of the H − Θ−3E clade contain a unique, highly conserved ORF located within the E1 + 1 ORF (light blue bars in Fig. 3A), known as E9. The E9 protein is 144–200 amino acids in length. Importantly, E9 is located within the N-terminal regulatory domain of E1. Unlike the other regions of the E1 protein, this N-terminal regulatory domain is relatively variable (Fig. 3A;Bergvall et al. 2013), suggesting that E9 is not simply the result of sequence constraints dictated by the E1 ORF. Importantly, FgPV1 and the other non-H − Θ−3E viruses do not contain an E9-like ORF.

### 3.5 The use of ‘molecular morphology’ provides support for a single topology

In addition to the presence of a unique E9 within the members of the H–Θ-3E clade, all sauropsid papillomaviruses have several unique features that may help with anchoring the evolutionary history of these viruses. Unlike the prototypical ‘mammalian virus’ E6 protein that consists of two 70-residue zinc-binding repeats (Vande Pol and Klingelhutz 2013), the sauropsid papillomaviruses contain an E6 protein that consists of a single zinc-binding domain (Van Doorslaer et al. 2009). Since all sauropsid papillomaviruses have this feature, the single-domain E6 was likely present in the most recent common ancestor of the (known) sauropsid papillomaviruses, implying it was present before the acquisition of the E9 protein (blue branch indicated by ‘1’ in Fig. 1). Interestingly, PePV1 (one of the H − Θ−3E viruses) appears to have lost the E6 ORF (purple branch labeled ‘2’ in Fig. 1).

Furthermore, the typical ‘mammalian virus’ E7 consists of a natively unfolded N-terminal region followed by a zinc-binding domain, which folds as an obligate homodimer (Roman and Munger 2013). The avian E7 proteins contain an extended unfolded N terminus, while the zinc-binding domain is relatively smaller when compared to the mammalian E7 (Van Doorslaer et al. 2009). Remarkably, the turtle E7-like proteins have completely lost the N-terminal region and acquired a shorter C-terminal extension (Fig. 3B; orange branch labeled ‘3’ in Fig. 1). Despite the loss of the CR1 and CR2 motifs, the turtle E7 zinc binding motif is more closely related to the H − Θ−3E papillomaviruses than to the FgPV1 E7 (Fig. 3C).

The most parsimonious explanation suggests that the last common ancestor of FgPV1 and the sauropsid papillomaviruses contained a single domain E6, and an E7 protein consisting of CR1, CR2, and a relatively short zinc binding motif. Following the divergence of the turtle and the H − Θ−3E papillomaviruses, the avian papillomaviruses gained an E9 ORF, while the turtle papillomaviruses lost the CR1-CR2 motifs typical of E7. In addition, the duplication event giving rise to the prototypical E6 and the expansion of the E7 zinc binding motif must have occurred specifically on the clade giving rise to the mammalian papillomaviruses. This scenario can be traced along the branches of the ML solution tree (Fig. 2). Despite being statistically equivalent, the other topologies tested above would need to invoke additional events to explain the evolution of the E6, E7, and E9 proteins.

Combining the inconclusive phylogenetic clustering with this additional molecular morphology data enables us to hypothesize that FgPV1 diverged from the other sauropsid papillomaviruses before the avian-turtle host animal diverged. This conclusion is further supported by the observation that FgPV1 lesions are uniquely associated with cartilage, while all other known papillomaviruses are primarily epithelial trophic (Gaynor et al. 2015).

### 3.6 Divergence of FgPV1 from the other sauropsid viruses may coincide with the paleozoic–mesozoic transition

**Figure 4.**
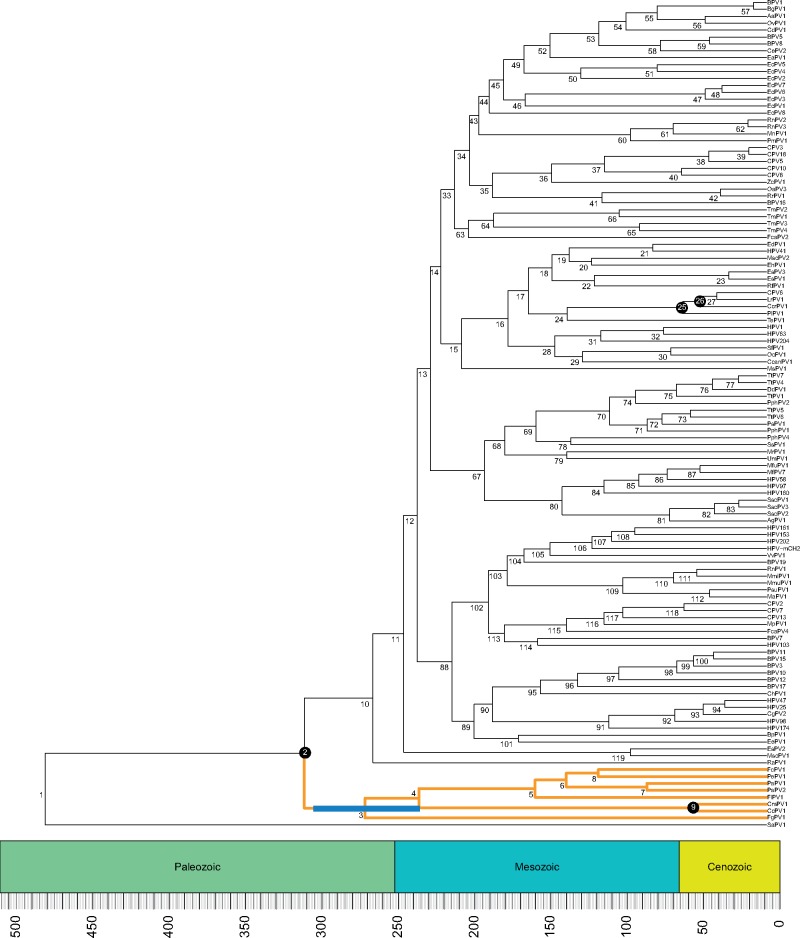
Time calibrated phylogenetic tree of 120 papillomaviruses. Host divergence time estimates were used to time calibrate the papillomavirus tree using BEAST2 (Bouckaert et al. 2014). The nodes indicated with the black circle correspond to the calibration nodes. Nodes were calibrated using a lognormal prior (see Supplementary Table S1 for exact values). The blue bar around node 3 indicates the 95 per cent highest probability density associated with the time estimate for the FgPV1 divergence. Support values and exact estimates (with 95% highest posterior density) for each numbered node are presented in Supplementary Table S1. Sauropsid papillomavirus branches are highlighted in orange and bold. The tree was constructed in BEAST2. Tree and geological column were visualized using the APE (Paradis et al. 2004), phyloch (available from http://www.christophheibl.de/Rpackages.html), and strap (Bell and Lloyd 2014) packages within R. The scale bar indicates millions of years before the present.

**Figure 5.**
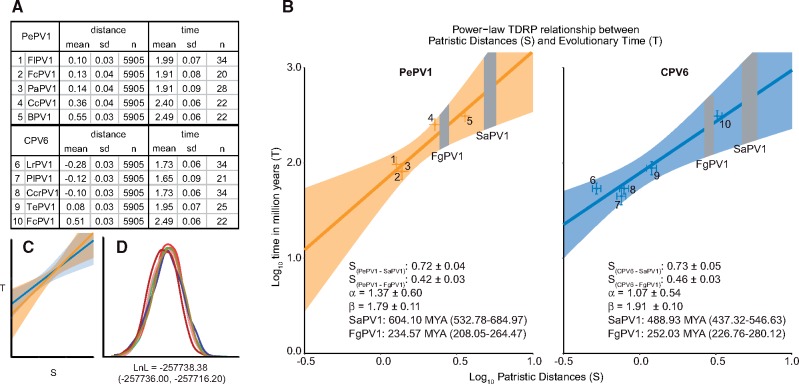
Evolutionary timescale of FgPV1 estimated by using the power-law rate-decay model. (A) The power-law rate-decay model (T = αS^β^) accounting for the time dependent rate phenomenon requires the patristic distance between different viruses (S) and the associated host divergence times (T) (Aiewsakun and Katzourakis 2017). To account for phylogenetic uncertainty, the post burn in posterior tree sample of three independent MrBayes (Huelsenbeck and Ronquist 2001; Ronquist and Huelsenbeck 2003; Ronquist et al. 2012) runs was used as the basis to calculate S (n_trees_=5905). S values were derived by back calculating from CPV6 and PePV1. T estimates for host species pairs were downloaded from www.timetree.org (Hedges et al. 2006; Hedges et al. 2015). Individual S and T values were log_10_ transformed, the mean and SD is shown. (B) S and T mean values with associated standard deviation were plotted. The numbers by the data points refer back to panel (A). The gray bars show the associated error for the FgPV1 and SaPV1 S estimates. Deming regression was used to obtain mean (±SD) values for the alpha and beta parameters. To obtain real time estimates, the reverse log of the solved values is reported (mean with 95% confidence interval). The regression line and associated confidence interval were drawn using regular linear regression, and is for illustrative purposes only. (C) Overlay of the curves in B showing overlap of derived estimates. (D) Posterior distribution of log likelihood values showing that the three separate MrBayes analyses converged on the same solution.

We estimated the divergence time between FgPV1 and the other Sauropsid papillomaviruses. The time-calibrated phylogenetic tree (Fig. 4) was calibrated using host divergence estimates. The most recent common ancestor (MRCA) of extant amniotes (node 2; 312 MYA (297–326 MYA)) and turtles (node 9; 47.5 MYA (30.5-62.8 MYA)), as well as the divergence estimates for the MRCA of raccoons and dogs (node 25; 54 MYA (52–57 MYA)), and hyenas and dogs (node 26; 40 MYA (33–46 MYA)) were used. Calibration means and 95 per cent confidence interval were downloaded from TimeTree (www.timetree.org; Hedges et al. 2006; Hedges et al. 2015). This analysis dates the divergence of FgPV1 to about 262 MYA (95% highest probability density 228–294 MYA). Interestingly, this analysis dates the root of phylogenetic tree around 481 MYA (326–656 MYA). However, while the older divergence dates matched the hosts fairly well, this analysis showed that many of the more recent viral divergence estimates are significantly older than what has been shown for the associated hosts. It has been shown that evolutionary rate estimates depend on the age of the nodes used as calibration points (Duchene et al. 2014a,b; Aiewsakun and Katzourakis 2015). It was recently suggested that this time-dependent rate phenomenon (TDRP) could be modeled using a power-law decay function (Aiewsakun and Katzourakis 2015, 2017). Therefore, the relationship between the Patristic Distance (S) and evolutionary timescales (T) should also follow the same power-law function. To correct for TDRP, we constructed a model for mammalian papillomaviruses by tracing the canine CPV6 backward in time (Fig. 5A). CPV6 and its close relatives belong to the *Gammapapillomaviridae*, a genus that has previously been shown to share a co-speciation history with their mammalian hosts (Rector et al. 2007). Similarly, a TDRP model was generated by calculating backward from the parrot virus PePV1 (Fig. 5A). Each approach provided us with five pairs of S and T estimates we used for model construction (Fig. 5B). Importantly, both TDRP models had overlapping error, suggesting that they give similar solutions (Fig. 5C). We then extrapolated both models separately to estimate the timescale of FgPV1 divergence based on its patristic distance estimate (S). By assuming that viruses infecting modern-day mammals and birds share similar TDRP dynamics as their ancestors while incorporating phylogenetic uncertainty, the FgPV1 divergence event was inferred to have occurred around between 230 and 250 MYA (PePV1: 235 MYA (208–264 MYA), CPV6: 252 MYA (227–280 MYA)). Both the BEAST and TDRP derived approaches provide similar estimates (Table 3). Collectively, these approaches estimate the niche adaptation event associated with the divergence of FgPV1 to have occurred around 250 MYA (Table 3), during the Paleozoic-Mesozoic transition. Furthermore, these approaches date the root of the papillomavirus tree between 400 and 600 MYA (Table 3).

**Table 3.** Point estimates and associated 95 per cent highest probability density interval.

	BEAST analysis		TDRP analysis			
			PePV1		CPV6	
	Mean	95% HPD	Mean	95% HPD	Mean	95% HPD
FgPV1	262	228–294	235	208–264	252	227–280
root	481	326–656	604	533–685	489	437–547

95% HPD: 95 per cent highest probability density interval.

## 4. Discussion

We identified a novel papillomavirus (PaPV2) isolated from a 16-year-old female Adélie penguin. This novel virus shares 62.5 per cent nucleotide identity across the L1 ORF when compared to its closest neighbor, PaPV1 (Table 1). This suggests that PaPV1 and PaPV2 (both isolated from Adélie penguin samples at Cape Crozier, Ross Island, Antarctica) likely belong to the genus *Treisepsilonpapillomavirus*. There are roughly twice as many species of birds compared to mammals (9956 versus 5416), yet only six papillomaviruses have been described associated with avian hosts (Adélie penguin, African Grey parrot, common chaffinch, northern fulmar, and yellow-necked francolin). Despite only having isolated a few non-mammalian viruses, these viruses have some unique features not shared with the mammalian viruses, suggesting that it is worthwhile to expand our understanding of non-mammalian papillomavirus genomes.

Interestingly, FgPV1, a virus isolated from a Northern fulmar does not contain many features shared by the other sauropsid viruses. Indeed, based on phylogenetic hypothesis testing, we were not able to pinpoint the exact evolutionary history of FgPV1. As in most biological systems, strict co-evolution of host and species does not fully explain the papillomavirus evolutionary history. However, a model based on niche adaptation near the root of the phylogenetic tree, followed by host-virus co-speciation has been proposed (Shah et al. 2010; Gottschling et al. 2011; Van Doorslaer 2013). Indeed, a sauropsid papillomavirus phylogeny corresponding to strict co-evolution was rejected (Table 2), suggesting a possible role for niche adaptation. However, since papillomaviruses typically have very similar genomic organization and infect epithelial cells, it has been hard to provide evidence for these niche switches as they are likely very subtle changes. We show that the use of ‘molecular morphological’ information allows us to more precisely determine the phylogenetic position of FgPV1 and more importantly provide evidence for such a niche adaptation event. Also, the unique plasticity of the early genes provides us with insights into the evolutionary history of the viral oncogenes.

The prototypical (mammalian virus) E6 protein consist of two highly conserved Zinc finger motifs (CxxC-x_29_-CxxC; where C is a cysteine and ‘x’ can be any amino acid) (Zanier et al. 2013). However, the E6 proteins of all known sauropsid papillomaviruses contain only one such motif. Since the fish papillomavirus (SaPV1) does not contain any such motif (Gaynor et al. 2015), it is likely that the most recent common ancestor of the mammalian and sauropsid papillomaviruses had a single motif E6, which duplicated to give rise to the mammalian papillomavirus double Zinc finger motif in E6 (Van Doorslaer et al. 2009). The sauropsid (putative) E7 protein may be even more remarkable. As shown in Fig. 3, the sauropsid papillomaviruses express three distinct variants of this protein. While FgPV1 contains an E7 that is very similar to the mammalian E7 protein, the other bird viruses’ (‘H–Θ-3E’) E7 is more compact (Van Doorslaer et al. 2009). Remarkably, the two papillomaviruses associated with lesions on turtles have a unique E7 protein. Unlike the other sauropsid viruses, the turtle E7 protein completely lacks the non-folded CR1 and CR2 motifs typically observed in E7. For the mammalian viruses, these motifs have been shown to interact with many cellular partners (Roman and Munger 2013). For example, it is likely that these E7 proteins will not be able to bind to pocket family proteins (e.g. pRb), a function believed to be important for the (α-) papillomavirus lifecycle (Roman and Munger 2013). The E6 and E7 proteins of these sauropsid viruses seem to be highly plastic. Remarkably, compared to the core proteins (E1, E2, L1, and L2), the E6 and E7 proteins are significantly less variable, suggesting that these ‘adaptive’ proteins may be under less functional constraints.

In addition to encoding these sauropsid-specific E6 and E7 proteins, the ‘H–Θ-3E’ viruses may also contain a unique E9 ORF. This putative protein is encoded within the +1 ORF of E1 and is embedded within the N-terminal regulatory domain, a part of E1 that is relatively less conserved (Bergvall et al. 2013). This suggests that the evolutionary pressure to maintain the E9 protein is not (solely) due to constraints on the E1 protein. The mammalian viruses express a spliced transcript that encodes fusion protein that consists of a short peptide from an ORF designated E8 fused to the C-terminal domain of E2 (Jeckel et al. 2003). The resulting proteins (designated E8^E2) function as repressors of viral transcription and replication (Jeckel et al. 2003). Importantly, while the E8 exon of E8^E2 is located in the same general region of the E1 protein as E9, there are no other obvious similarities between both proteins, suggesting that both proteins have separate origins. It had been previously suggested that the N-terminal region of E1 may be a privileged segment of the protein more malleable to evolutionary innovation (Morin et al. 2011). While it is intriguing to note that a subset of bird papillomaviruses carry such an E9 ORF, in the absence of functional data it is hard to speculate about the functionality of this protein.

Importantly, MsPV1 (associated with a lesion on a python) does not cluster with any of the other known non-mammalian tropic papillomaviruses (Fig. 2). The phylogenetic position of this virus significantly complicates the co-speciation hypothesis (with or without niche adaptation) between papillomaviruses and their hosts. This dilemma could be explained by invoking a cross-species infection (Lange et al. 2011). However, it is believed that papillomaviruses are highly species specific. Until this branch of the phylogenetic tree becomes populated with more reptilian viruses, it is impossible to rule out alternative explanations (e.g. contamination of the python lesion with mammalian virus DNA).

Inclusion of ‘molecular morphological’ supports the ML solution reached by the evolutionary sequence analysis, i.e. FgPV1 speciated before the split between the turtle and bird viruses. Of note, FgPV1 viral particles were found in the cartilaginous tissue of a Northern fulmar’s foot (Gaynor et al. 2015). Since papillomaviruses are typically associated with epithelial tissues, FgPV1 may be the prototype of a new group of papillomaviruses able to replicate within cartilage, a dramatically different host tissue. To estimate the timing of this niche adaptation event, we constructed a traditional host constrained time tree for viral evolution. In addition, due to concerns about TDRP, we estimated the date using a complimentary approach that has been suggested to be less sensitive to TDRP (Aiewsakun and Katzourakis 2017). Using both approaches, we estimated divergence of FgPV1 to have occurred around 250 MYA ago. This overlaps with the Paleozoic-Mesozoic transition. Not only was this transition associated with mass extinction but also coincides with the rise of endothermia (Rey et al. 2017). The combination of mass extinctions and (molecular) changes associated with the ability regulate internal temperature may have provided the right evolutionary environment for this niche adaptation event to be successful. Based on our analysis, the ancestor of the amniote-associated viruses infected epithelial cells. Along the evolutionary path toward the sauropsid viruses, intra-host divergence (i.e. independent of host speciation) (Buck et al. 2016) created an ancestral virus capable of infecting a novel host niche (i.e. chondrocytes). This hypothesis suggests that there should be several (if not hundreds) papillomaviruses with a tropism for cartilage. In addition, this analysis estimates that papillomaviruses have a marine origin and may have evolved along with their jawed vertebrate hosts. This is reminiscent of the ancient marine origin of retroviruses (Ruboyianes and Worobey 2016; Aiewsakun and Katzourakis 2017;).

As part of this study we isolated a novel papillomavirus from Adélie penguins. Detailed sequence analysis of this novel virus and other non-mammalian papillomaviruses provided us with novel insights into the evolution of these viruses. We provide evidence for genomic plasticity (specifically within the viral E6 and E7 genes). Furthermore, we generated the testable hypothesis that viral niche adaptation gave rise to a clade of viruses with a tropism for cartilage. A concerted effort to identify additional, novel viruses will allow us to fine tune our hypothesis on genome plasticity and whether niche adaptation events similar to the one seen in the Norther Fulmar are more common.

## Supplementary Material

Supplementary Text

Supplementary Table 1
